# Next-generation sequencing reveals large connected networks of intra-host HCV variants

**DOI:** 10.1186/1471-2164-15-S5-S4

**Published:** 2014-07-14

**Authors:** David S Campo, Zoya Dimitrova, Lilian Yamasaki, Pavel Skums, Daryl TY Lau, Gilberto Vaughan, Joseph C Forbi, Chong-Gee Teo, Yury Khudyakov

**Affiliations:** 1 Division of Viral Hepatitis, Centers for Disease Control and Prevention, Atlanta, Georgia, USA; 2 Laboratory of Genomic Studies, Department of Biology, UNESP - São Paulo State University, Brazil; 3 Liver Center, Division of Gastroenterology, Beth Israel Deaconess Medical Center, Harvard Medical School, Massachusetts, USA

## Abstract

**Background:**

Next-generation sequencing (NGS) allows for sampling numerous viral variants from infected patients. This provides a novel opportunity to represent and study the mutational landscape of Hepatitis C Virus (HCV) within a single host.

**Results:**

Intra-host variants of the HCV E1/E2 region were extensively sampled from 58 chronically infected patients. After NGS error correction, the average number of reads and variants obtained from each sample were 3202 and 464, respectively. The distance between each pair of variants was calculated and networks were created for each patient, where each node is a variant and two nodes are connected by a link if the nucleotide distance between them is 1. The work focused on large components having > 5% of all reads, which in average account for 93.7% of all reads found in a patient.

The distance between any two variants calculated over the component correlated strongly with nucleotide distances (r = 0.9499; p = 0.0001), a better correlation than the one obtained with Neighbour-Joining trees (r = 0.7624; p = 0.0001). In each patient, components were well separated, with the average distance between (6.53%) being 10 times greater than within each component (0.68%). The ratio of nonsynonymous to synonymous changes was calculated and some patients (6.9%) showed a mixture of networks under strong negative and positive selection. All components were robust to *in silico *stochastic sampling; even after randomly removing 85% of all reads, the largest connected component in the new subsample still involved 82.4% of remaining nodes. *In vitro *sampling showed that 93.02% of components present in the original sample were also found in experimental replicas, with 81.6% of reads found in both. When syringe-sharing transmission events were simulated, 91.2% of all simulated transmission events seeded all components present in the source.

**Conclusions:**

Most intra-host variants are organized into distinct single-mutation components that are: well separated from each other, represent genetic distances between viral variants, robust to sampling, reproducible and likely seeded during transmission events. Facilitated by NGS, large components offer a novel evolutionary framework for genetic analysis of intra-host viral populations and understanding transmission, immune escape and drug resistance.

## Background

Hepatitis C virus (HCV) infects nearly 3% of the world's population and is a major cause of liver disease worldwide [1]. There is no vaccine against HCV and up to recently standard-of-care therapy involved the combined use of pegylated interferon (peg-IFN) and ribavirin (RBV). This combination therapy is expensive, effective in only 50%-60% of patients, and can be associated with frequent and serious adverse side effects in >75% of patients [2,3].

HCV is genetically very heterogeneous and classified into 7 genotypes and numerous subgenotypes [4]. HCV changes continuously during chronic infection, showing a complex dynamics of intra-host viral subpopulations [5]. The most studied HCV region is the hypervariable region 1 (HVR1) located at amino acid (aa) positions 384-410 in the structural protein E2. Sequence variation in HVR1 correlates with neutralization escape and is associated with viral persistence during chronic infection [6-11].

Recent advances in next-generation sequencing (NGS) methods allow for analysis of the unprecedented number of HVR1 sequence variants from infected patients and present a novel opportunity for understanding intra-host HCV evolution, drug resistance and immune escape [12]. HCV HVR1 variants sampled using NGS from infected individuals were studied here. We report, for the first time, that most variants are organized into large single-mutation connected components. This finding presents a novel framework for genetic analysis of intra-host HCV populations, relevant to the study of viral transmission, immune escape and drug resistance.

## Results

### One-step networks

**Figure 1:**
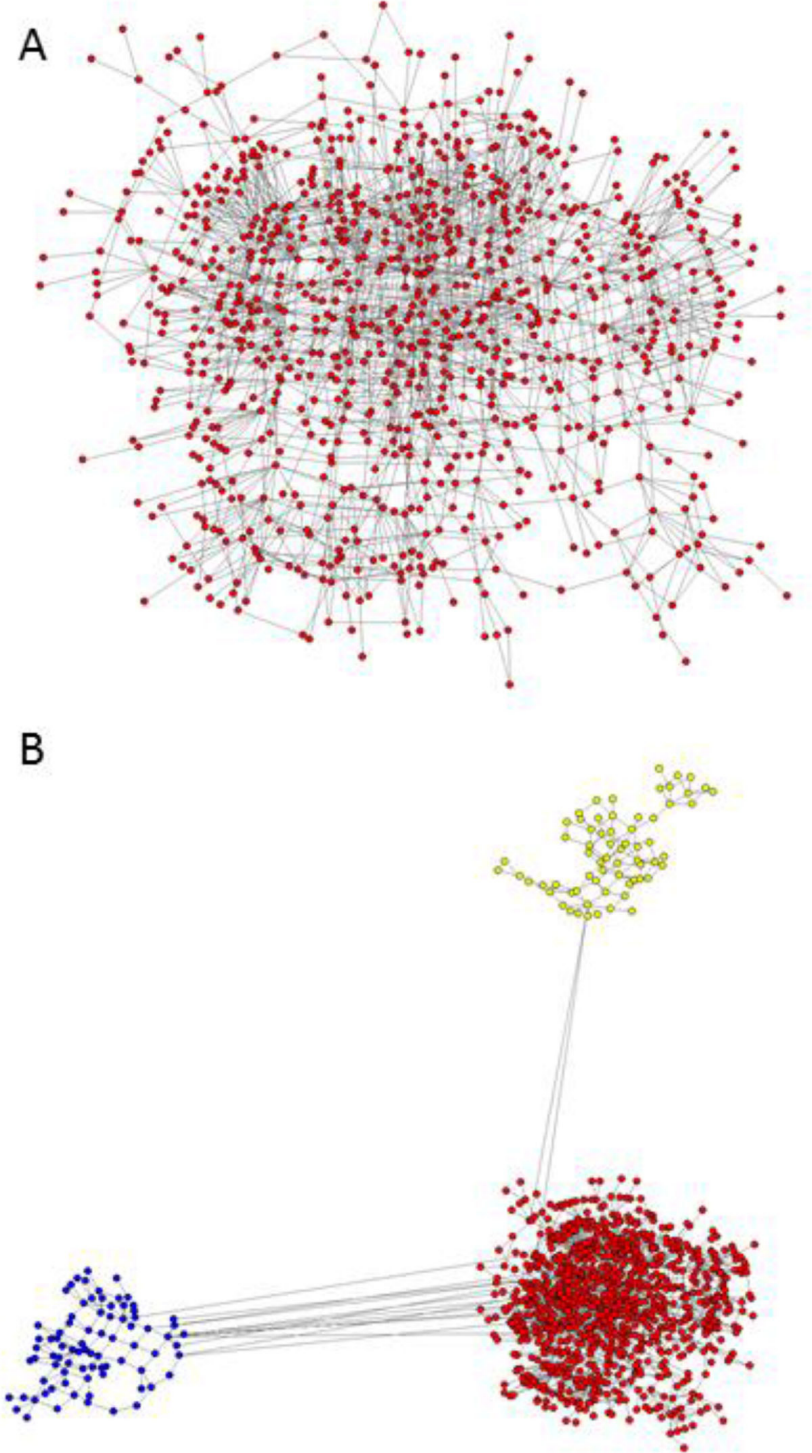
**One-step components of a single patient**. A) Largest one-step component of patient 14, where each node is a variant and two nodes are connected by a link if the nucleotide distance between them is 1. B) k-step network showing the three components of patient 14. Each one-step component is shown in a different colour and links among component connect the pairs of variants having the minimal distance found among components.

Intra-host variants of the E1/E2 region were extensively sampled from 58 patients chronically infected with HCV genotypes 1 or 3. After NGS sequencing error correction, the average number of reads and variants obtained from each sample were 3202.43 (Standard Error of the Mean, S.E.M. = 330.41) and 464 (S.E.M. = 79.98), respectively. In all patients, the average frequency of the major variant was 0.32 (S.E.M. = 0.03). The distance between each pair of variants was calculated and networks were created for each patient, where each node is a variant and two nodes are connected by a link if the nucleotide distance between them is 1. The subsequent research was focused only on large components, which were arbitrarily defined as encompassing >5% of all reads. This threshold was chosen because it defines a small number of large components that still account for most reads found in a patient (Mean = 93.7%, S.E.M. = 0.78). In average, a patient had 2.17 components (S.E.M. = 0.17), ranging from 1 to 5 in each patient; each component having in average 172.99 variants (S.E.M. = 20.96) and a total of 1414.85 reads (S.E.M. = 148.23) (Figure 1).

### Network topological analysis

All components showed a skewed degree distribution. Although not strictly following a power-law degree distribution, most nodes are incident only to one or two links. The shortest path distances among all pairs of sequences were estimated, and then the average shortest path distance for each component was calculated. Over all components, the average pairwise distance shortest path was 4.06 (S.E.M. = 0.14), whereas the diameter was 9.24 (S.E.M. = 0.41). Interestingly, the nodes in the component did cluster very little (as measured by clustering coefficient, average over all patients = 0.0071, S.E.M. = 0.002). A low clustering coefficient indicates the absence of triangles in the network. The only way to form a triangle is to have three sequences that are one mutation away from each other at the same nucleotide position. The absence of this network motif simply indicates that close sequences of this HCV genomic region often differ at distant positions, instead of differing in states of the same position.

**Figure 2:**
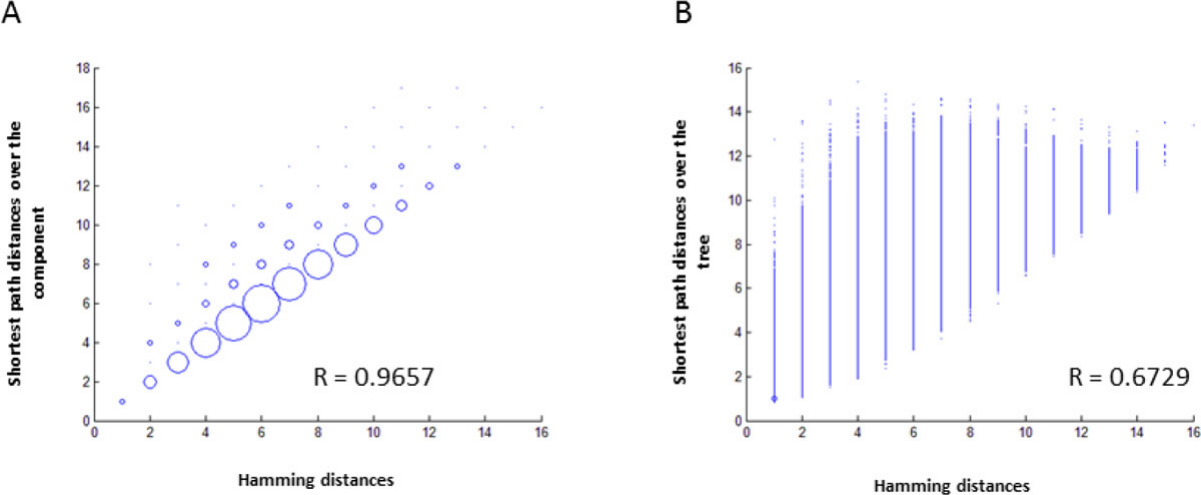
**Scatterplot of hamming distances among the variants of the largest component of patient 14**. A) Scatterplot of hamming distances (x-axis) and shortest path distances over the one-step component (y-axis). B) Scatterplot of hamming distances (x-axis) and shortest path distances over the Neighbor-Joining tree (y-axis). The R value is the correlation among the two distance matrices. The size of the circles indicate the number of points (pairwise comparisons) in those coordinates.

The component distances highly correlated (average r = 0.9499; S.E.M. = 0.005; all with p = 0.0001) with the Hamming distances between sequences in all patients. When patristic distances were calculated over a Neighbor-Joining tree for sequences included in each component, the correlation between the original Hamming distances and tree-based distances was lower in all cases, with an average of 0.7624 (S.E.M. = 0.01; all with a p = 0.0001). These results indicate that the distances over the component represented the true distances more accurately than the distances over the tree (Figure 2).

### *In silico *sampling robustness

**Figure 3:**
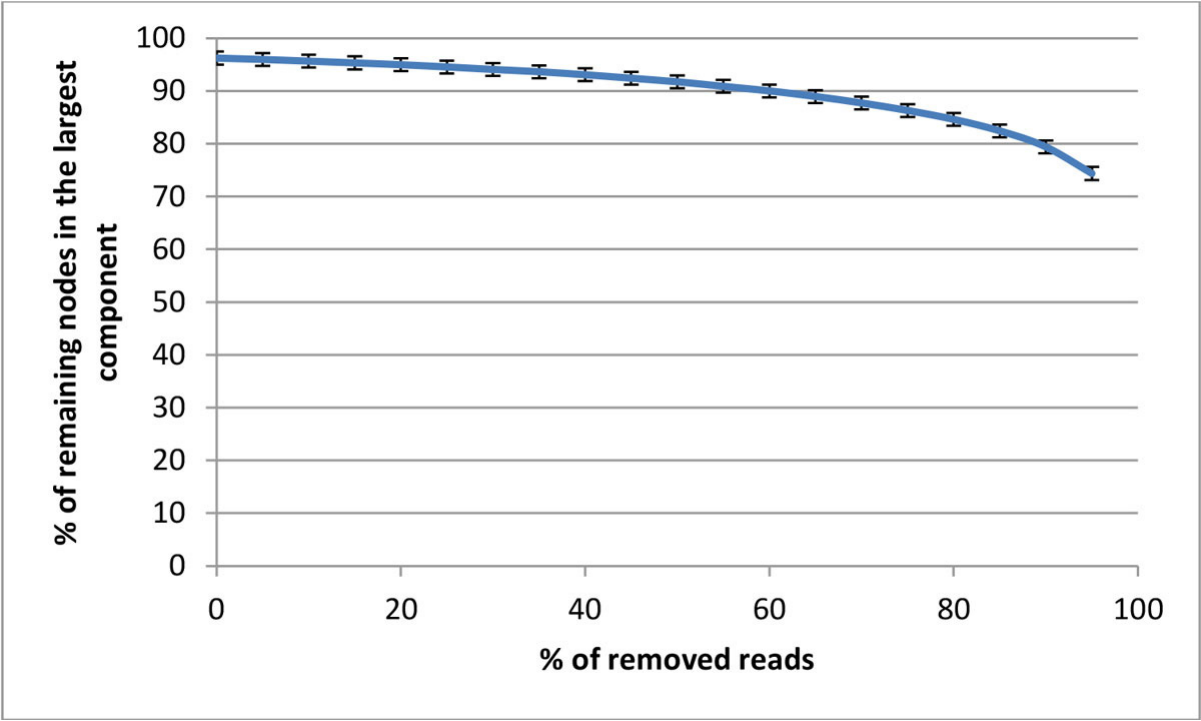
***In silico *sampling robustness of components**. Mean over 1000 samples for each component and sampling level, with bars indicating the 99% confidence interval for the mean. The x-axis represents the percentage of reads removed from the original dataset. The y-axis represents the percentage of nodes in the subsample that are part of the largest component.

The robustness of each component to sampling bias was evaluated by measuring the decline in component connectivity with reduction of the sample size. All components were extraordinarily robust to this type of stochastic sampling; even after randomly removing 85% of all reads, the largest connected component in the new subsample still involved 82.42% of the remaining nodes (S.E.M. = 1.69%) (Figure 3).

### *In vitro *reproducibility

**Figure 4:**
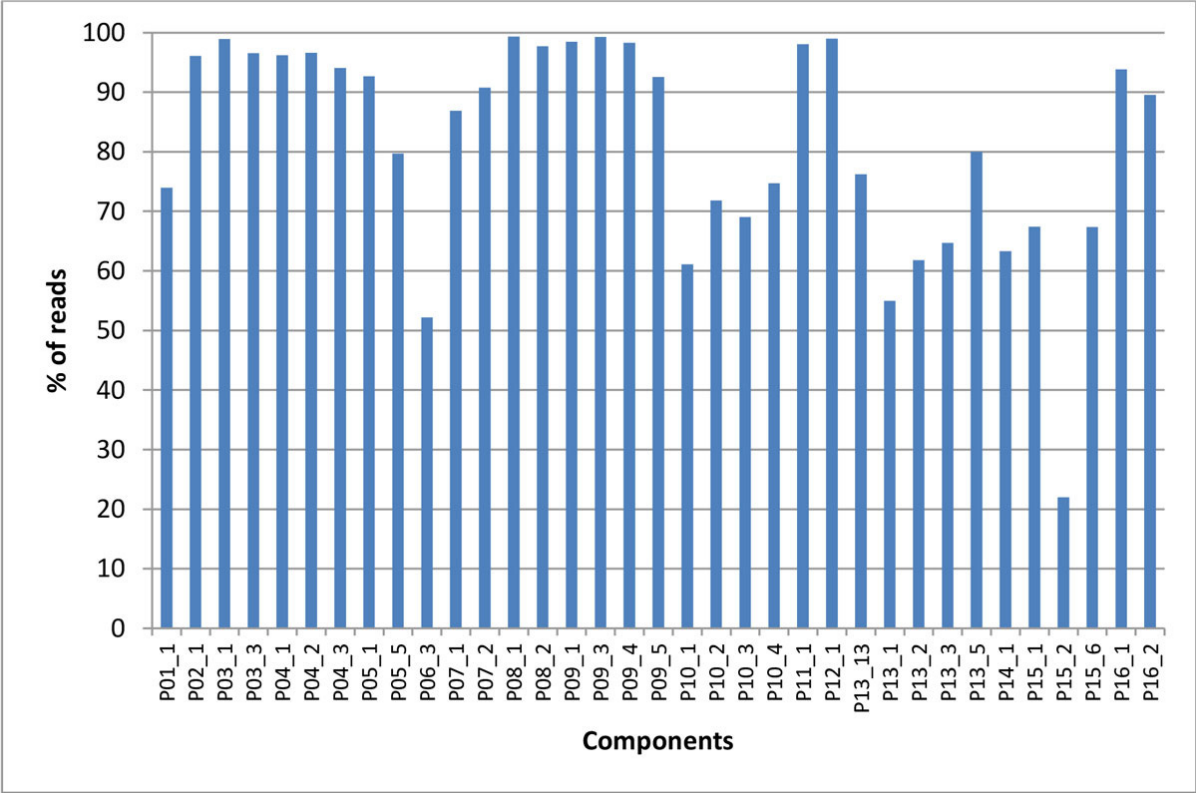
**Component overlap**. The x-axis represents the 35 components found in the first and second experimental sample. The y-axis shows the fraction of reads found in the first sample that were also found in the second sample.

For 16 of the patients, a second sample which was taken one hour after the first one was available, providing the opportunity to test whether the components found in the first sample were also present in the second sample, an indication of experimental reproducibility. The results showed that 93.02% of the components present in the first samples were found in the repeat samples. For these components found in both samples, we calculated the fraction of reads found in the first sample that were also found in the second sample, obtaining an average over all components of 81.57% (S.E.M. = 18.20) (Figure 4). This result suggests that intra-host subpopulations modelled with components are experimentally stable. All components missing in the second sample had an initial frequency between 5.2% and 5.4% of all reads, indicating that the 5% arbitrary frequency cut-off chosen in this work for a component to qualify as "large" is near the limit of experimental replication.

### Simulated transmission events

We simulated unsafe injection-related transmission events using estimates of the number of virions (n = 24) that may establish productive infection of liver from contaminated syringes (Methods section). In order to simulate the transmission event, a random subsample (recipient) of only 24 reads was taken from the reads of a given patient (source). For each recipient sample, we tested the number of components from the source represented by the sampled reads. Importantly, we found that 91.20% of all simulated transmission events seeded all components present in the source (S.E.M. = 0.57%), indicating that viral variants that have established infection in a recipient after transmission likely represent all components existing in the source, thus allowing for the potential reconstitution of these components from a small but heterogeneous founder population.

### Differences among components

**Figure 5:**
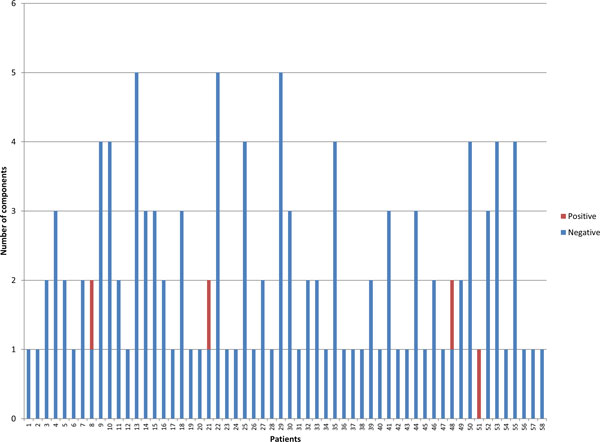
**Natural selection of components**. The x-axis represents the 58 patients that were analysed. The y-axis represents the number of components. The blue colour shows the components with dN/dS ratio lower than 1, whereas the red colour indicates the components with dN/dS ratio higher than 1.

The components of each patient were well separated, with the average Hamming distance between their members (average = 6.53%; S.E.M. = 0.18%) being ~10 times greater than within each component (average = 0.68%; S.E.M. = 0.04%). For each component, the overall ratio of nonsynonymous (dN) to synonymous changes (dN) was calculated. Most of the patients (93.10%) harboured only negatively selected components, whereas 1.72% harboured only positively selected components. However, 3 patients (5.17%) had a mixture of components under negative or positive selection (Figure 5).

## Discussion

RNA viruses exhibit extremely high mutation rates and given the large population sizes observed in both experimental and natural infections with these viruses, every possible point mutation and many double-mutation combinations could theoretically be generated during each replication cycle within a population. The mutability of each viral genome means that RNA virus populations exist as dynamic mutant networks in which sequences are continuously diversified and regenerated by mutation of related sequences, often known as a quasispecies [13]. Connecting viral variants that differ in one mutation provides a natural framework to organize the heterogeneous population of viral variants. Although this has been achieved on theoretical landscapes (e.g. RNA secondary structures), here we have built these one-step networks for the first time using viral experimental data, a development just recently enabled by the NGS technology.

Analyses conducted using *in silico *and experimental sampling showed significant robustness of the component to stochastic variation, implying that a true component will not be split into two due to poor sampling. Another advantage of the network framework is that several topological parameters can be calculated (e.g., degree distribution, eigenvector centralities, modularity, motif distribution) for a given patient and then used to test for differences among different phenotype groupings (e.g., acute vs. chronic patients, or responders vs. non-responders of antiviral therapy). Although this topological comparison was not performed in the present research, this network framework provides a new potential tool for comparing viral populations.

The simulated transmission events may seem too simplified, as selective pressure in the new host environment will likely be different, affecting intra-host evolution in a different way. However, the simulation does not deal with the survival of those seeds, only with their likelihood of being transmitted. This is in agreement with many reports on HCV outbreaks, where it is very common that newly infected cases share variants and are genetically very close to the transmission source [14]. Based on this, the purpose of the simulation was to show a very simple phenomenon: if transmission is purely stochastic (there are not differences in transmissibility among variants), then the variants present in the source will be transmitted to the incident case in proportion to their frequency. As was shown in the transmission simulation experiments, the high frequency of components allows a small random sample of 20-30 variants to represent with high probability all of these components in each intra-host viral population. This important property facilitates reconstitution of the same component composition of intra-host viral population from the small number of founder variants transmitted from one host to another.

The level of genetic separation among large components indicates that exploration of all the components in each infected host likely cannot be accomplished via a single mutation starting from a single viral variant. The existence of several subpopulations organized in one-step components in each infected individual studied here suggests that the founder viral population was complex, in agreement with recent findings [15]. The data obtained in this study indicate that the mechanism of stochastic sampling is compatible with the complex founder model and further experiments are underway to resolve this issue.

HCV changes continuously during chronic infection, showing a complex dynamics of intra-host viral subpopulations [5]. Large components of HCV variants obtained from follow-up samples could be used to define viral subpopulations, whose shifts in frequency over time can be tracked to pinpoint the sources of drug resistance or immune escape. The one-step connection provides a simple biological criterion for identifying the genetic structure of viral subpopulations. When sampling of intra-host viral variants is done using NGS, the one-step components can be generated for clustering closely related variants without any assumption on their ancestral origin. The genetic space occupied by the variants of a given component will likely be explored very rapidly via single mutations starting from any variant in each component. The estimation of the amount of time needed for this exploration should take into account the kinetic parameters of HCV infection (population size, mutation rate, doubling time, liver volume, etc.). These are currently being investigated in our laboratory. Given that some patients harbour a mixture of positively and negatively selected components, the subpopulations defined by components may experience different selection pressures, accounting for the frequency shifts observed during intra-host evolution in some patients [5].

The distances calculated over the component were found to be almost identical to the Hamming distances calculated for all variants, which allows for better visualizing local and global relationships among sequences and among clusters. It must be noted that other popular representations of the distance matrix, such as hierarchical bifurcating trees or Multi-Dimensional Scaling, reduce dimensionality of data at the cost of accuracy of distance representation among genetic variants. We have shown here that patristic distances calculated over the Neighbor-Joining tree have a significantly lower correlation with the distance matrix than the one-step components. A good phylogenetic tree presents a single most likely history of substitutions, all of which collectively generated the dataset. However, the key point of our work is that a single history is an over-simplification when the whole quasispecies network is continuously generated over multiple parallel paths. This network setting fits better the highly dynamic process which is the result of HCV's large population size, high mutation rate and fast doubling time.

Although NGS has been instrumental in the acquisition of a massive number of genomic sequences, the contribution of these sequences to understanding intra-host viral evolution have been modest, considering the enormous amount of genetic information generated by the technology [12]. This paradox can, in part, be explained by the shortage of computational methods for verifying the accuracy of generated sequences and accommodating large datasets. However, extensive sequencing of intra-host viral variants offers also novel opportunities for analysing genetic information, which are not available for small samples of sequences. One such approach was developed in this study. We have shown that one-step components represent accurately genetic relationships among the HCV HVR1 variants extensively sampled by NGS from infected individuals. This likely reflects sampling of numerous intermediates and/or co-existent ancestors, which allows the parsimonious reconstruction of the evolutionary process responsible for generation of the intra-host viral population.

## Conclusions

The majority of intra-host HVR1 variants form large connected components in infected patients. These large components preserve true distances among variants, are well separated from each other, are robust to sampling, and are likely seeded during transmission events. Large components identified through NGS-facilitated deep sampling offer a novel evolutionary framework for genetic analysis of intra-host HCV populations to characterise viral factors relevant to transmission, immune escape and drug-resistance.

## Methods

### Samples

Intra-host HCV variants were extensively sampled from 58 chronically infected patients with genotypes 1 or 3. Ethical review and informed consent were granted by the institutional review boards of Beth Israel Deaconess Medical Center and the Centers for Disease Control and Prevention.

### Nucleic acid extraction

Total nucleic acids from the specimens were extracted from serum using the Roche MagNA Pure LC instrument and the MagNA Pure LC Total Nucleic Acid Isolation Kit (Roche Diagnostics, Mannheim, Germany). RNA was precipitated and reverse-transcribed using both random and specific primers as previously described [16]. PCR quantification was conducted by the COBAS AmpliPrep/COBAS Taq-Man HCV Test (Roche Diagnostics, Mannheim, Germany), and HCV genotype determined using the VERSANT HCV Genotype 2.0 Assay (LiPA) (Innogenetics NV, Gent, Belgium).

### HVR1 cDNA amplification

The E1/E2 junction of the HCV genome (309 nt), which contains the HVR1 region, was amplified using our nested PCR protocol as previously described [16]. The amplicons generated during first-round PCR were used as templates for nested PCR using hybrid primers composed of primer adaptors, multiple identifiers and specific sequences complementary to the HCV genome. This strategy allowed for multiplexing and downstream pyrosequencing. Resulting amplicons were quantified using the Picogreen kit (Invitrogen, Carlsbad, CA). Integrity of each fragment was evaluated using Bioanalyzer 2100 (Agilent, Santa Clara, CA).

### Next Generation Sequencing (NGS)

PCR products were pooled and subjected to pyrosequencing using the GS FLX System and the GS FLX Titanium Sequencing Kit (454 Life Sciences, Roche, Branford, CT). Low-quality reads were removed using the GS Run Processor v2.3 (Roche). Initial reads were processed by matching to the corresponding identifier. The 454 files were processed using the error correction algorithms KEC and ET [17], which have been validated to be highly accurate in finding true haplotypes, estimate their frequencies and remove false ones. The error-corrected files were aligned using Muscle [18] and the HVR1 sequences clipped to 303 bp.

### Nucleotide sequences

The E1/E2 sequences produced in this study can be found in the following link: http://alan.cs.gsu.edu/~skumsp/BMC_genomics.fas. The name of the sequence includes the patient name, a unique identifier, the genotype and the absolute frequency (number of reads).

### One-step network

The distance between each pair of variants was calculated and networks were created for each patient, where each node is a variant and two nodes are connected by a link if the nucleotide distance between them is 1. The present work was focused on large components having more than 5% of all reads. Most analyses in this paper were performed with MATLAB R2011b (The MathWorks, Inc., Natick, MA). Network visualization was achieved with the programs Pajek [19] and Gephi [20].

### k-step network

In order to visualize how distant or similar the one-step components are, a simple method for parsimoniously connect the components was devised. The k-step network method consist of the following steps: (i) calculation of the Hamming distance between each pair of variants belonging to different one-step components; (ii) determination of the minimum among these distances; and (iii) establishment of a link among the variants belonging to different one-step components that have this minimal distance.

### Sampling robustness

For each component, bootstrap samples with replacement were created. Briefly, a number of reads equal to a defined percentage of the original number of reads was taken randomly from the original component. The procedure was repeated at different sampling levels, with decreasing number of reads in 10% intervals. For each component and sampling level, the procedure was repeated 1000 times. For each sample, the one-step network was built and the size of the largest component was calculated. An average over all samples and a 99% confidence interval were calculated.

### Simulation of transmission events

The average needle-sharing event would transmit 24 virions, a number based on the following estimates: 2 uL of blood contaminates a syringE [21]; 1 × 10^7 ^virions per 1000uL of blood [22]; the infection rate is 1.5 × 10^-6 ^per mL/virion/day [23]; and an average liver volume of 1400 mL [24]. In order to simulate the transmission event, we took all the reads of patient (source) and created a subsample with replacement made of 24 reads (recipient), a process that was repeated 1000 times per patient. For each recipient sample, we tested how many components present in the source were represented by one or more reads.

### Evidence of selection

For each component, we calculated the overall ratio of nonsynonymous substitutions on nonsynonymous sites (dN) over the synonymous substitutions on synonymous sites (dS) with a modified version of the single-likelihood ancestor counting (SLAC) algorithm [25]. In our approach, rather than counting the number of substitutions for each codon over the branches of the phylogenetic tree, the number of substitutions was counted over all the edges of the one-step component.

## Competing interests

The authors declare that they have no competing interests.

## Authors' contributions

DSC and YK designed the project; LY, DYTL and CGT procured samples; LY, GV and JCF performed laboratory experiments; DSC, ZD and PS analyzed data; DSC and YK wrote the manuscript; all authors revised the manuscript.

## Declarations

Publication costs for this article were funded by the corresponding author's institution, the Centers for Disease Control and Prevention.

This article has been published as part of *BMC Genomics *Volume 15 Supplement 5, 2014: Selected articles from the Third IEEE International Conference on Computational Advances in Bio and Medical Sciences (ICCABS 2013): Genomics. The full contents of the supplement are available online at http://www.biomedcentral.com/bmcgenomics/supplements/15/S5.
